# Compromised Nutritional Status as a Risk Factor for the Incidence of Nosocomial Infections

**DOI:** 10.7759/cureus.46502

**Published:** 2023-10-04

**Authors:** Snigdha Gupta, Himangi Lubree, Sonali Sanghavi

**Affiliations:** 1 Food and Nutrition, Savitribai Phule Pune University, King Edward Memorial Hospital Research Centre, Pune, IND; 2 Nutrition, King Edward Memorial Hospital Research Centre, Vadu Rural Health Program, Pune, IND; 3 Microbiology, King Edward Memorial Hospital Research Centre, Pune, IND

**Keywords:** serum albumin, subjective global assessment, nutritional status, nosocomial infections, risk factors

## Abstract

Background

Poor nutritional status may lead to longer hospital stays, increased mortality and morbidity, increased cost, and higher suffering. Nosocomial infections (NI) are a global health concern, and several risk factors are associated with their higher incidence. This study aimed to reveal that compromised nutritional status is one of the risk factors for developing NIs.

Methodology

The study was conducted in a tertiary care hospital in Pune, India. This was a prospective cohort study with a sample size of 200 hospitalized participants. Data collection was based on standard tools and structured forms which had two parts. In the first part, the assessment of nutritional status was done for which patients were categorized into two groups, namely, well-nourished and undernourished. Additionally, biochemical parameters (serum albumin) were also assessed. The second part included a follow-up of participants to evaluate the development of NIs including their laboratory investigation. Results were analyzed statistically using R software.

Results

Among 200 participants, 60 were female, of whom 15% developed NIs. Of the 140 males, 8% had NIs. Among 200 participants, 101 (51%) were well-nourished, of whom two (2%) developed NIs. Of the 99 (49%) undernourished participants, 18 (18%) had NIs. Those who were undernourished (univariate relative risk = 6.10, 95% confidence interval) were more prone to developing NIs compared to the well-nourished group.

Conclusions

NIs are widespread globally but are less studied and given less emphasis in developing countries. This study reports various types of NIs along with their incidence in well-nourished and undernourished groups. The incidence of NI observed in this study may reflect the higher severity of illness, age, poor nutritional status, and longer hospital stays. Identifying risk factors that can contribute to developing NI may help in their prevention by maximizing patient safety.

## Introduction

Globally, many patients are affected by nosocomial infections (NIs) which are associated with an increase in mortality rate and cost burden. According to the estimated report by the World Health Organization (WHO), among all hospitalized patients, 15% are affected by NIs [1].

The current nutritional status of an individual depends on previous nutritional status as it is reflected in terms of underlying disease conditions, deficiencies, and physical or mental wellness. Deficiency of any nutrients may lead to poor health and less resistance to infections. In hospitalized patients, it is more important to check their nutritional status as its deterioration can lead to other complications. Iron deficiency and protein energy malnourishment are more common in hospitalized patients and are of the greatest concern to public health [2]. To assess nutritional status many nutritional parameters are considered such as anthropometry, several nutritional screening tools, biochemical parameters, and certain nutritional markers.

Poor nutritional status may lead to longer hospital stays, increased mortality and morbidity, increased cost, increased physical or mental suffering, and loss of wages. According to studies, patients with poor nutritional status have more healthcare-associated infections than those with normal nutritional status [3].

A study by Nejad et al. [4] reported that the prevalence of NIs is high in high-income countries (3.5% and 12%) and varies between 5.7% and 19.1% in middle and low-income countries whereas neonates are three to 20 times more prone to it.

This study aims to assess whether nutritional status can be one of the risk factors for the development of NIs along with other confounding factors such as age and comorbidities. Early intervention can help reduce the risk of NIs and would be more helpful in the treatment of patients once its targeted risk factors are identified.

## Materials and methods

Study patients

This study was conducted at King Edward Memorial Hospital, Pune. A total of 200 cases with no NIs at the time of admission were studied from 2021 to 2022. Patients who were admitted to the hospital and later developed infections with hospital stays of more than 48 hours were included. Patients showing signs and symptoms of infection at the time of admission, those transferred from another hospital within the preceding 30 days from the current admission, and those with a history of medical procedures/invasive procedures within 48 hours of admission were excluded.

Study design and methods

A cohort study was conducted to evaluate the nutritional status of patients. A standard and validated tool, i.e., subjective global assessment, was modified and used at the time of admission. This tool had components such as weight change, dietary intake, gastrointestinal symptoms, functional capacity, metabolic stress, and physical assessments based on which patients were categorized into well-nourished and malnourished groups. Furthermore, serum samples of participants obtained at the time of admission were collected from hospital laboratories for assessing serum albumin levels as it reflects the changes in the nutritional state.

For confirmed cases of NIs, a combination of clinical features and laboratory investigations were recorded. Clinical features include signs and symptoms of respiratory tract infections, urinary tract infections, surgical site infections, bloodstream infections, and any other NIs. Laboratory investigations included blood tests, routine urine tests, diagnostic tests (X-rays), microbiological culture sensitivity tests, and clinical samples. To determine the incidence of NIs patients were followed up for 30 days or until their discharge. Written informed consent was taken from each participant.

Statistical analysis

R software was used for statistical analysis. Fisher’s exact test was used to determine the effect of exposure variables on an outcome for assessing infection status in categorical variables. The Kaplan-Meier curve was used to show the survival time for the patients in the context of developing NIs. Univariate Poisson regression analysis was used to describe the characteristics of each variable on an outcome. P-values <0.05 were considered statistically significant [5]. Relative risk (RR) of greater than 2.0 was considered clinically significant [6].

Ethical approval

This study was approved by the Ethics Committee of King Edward Memorial Hospital Research Centre, Pune (reference number: KEMHRC ID No. Ph. D. 26; dated 01/07/2020).

## Results

A total of 200 patients, admitted in different wards (medical, surgical, general, intensive care units) of King Edward Memorial Hospital, Pune were studied. Results comprised the following three parts: nutritional status of patients, distribution of NIs, and the association between nutritional status and NIs.

Categorization of nutritional status of patients

Among 200 participants, 60 were female and 140 were male, of whom nine (15%) and 11 (8%) developed NIs, respectively. The mean age was 53 years ranging between 39 and 65 years. In the adult group (≥18 years), five (4%) participants had NIs, and in the group of older adults (≥65 years), 15 (24%) participants had NIs (p < 0.003). Among 200 participants, 101 (51%) were well-nourished, of whom two (2%) developed nosocomial infections. Of the 99 (49%) undernourished patients, 18 (18%) had NIs (p < 0.001).

Out of 200 participants, 133 (67%) had a normal range of serum albumin, of whom nine (7%) had NIs, and 67 (34%) had abnormal albumin levels, of whom 11 (16%) had NIs. Out of 200 participants, 116 (58%) had no comorbidities, of whom seven (6%) had NIs; 16 (8%) had diabetes (DM) with no NIs; 26 (13%) had hypertension (HTN), of whom three (12%) had NIs; and 42 (21%) had both comorbidities, of whom 10 (24%) had NIs (Table 1).

**Table 1:** Nutritional status of patients. Note: P-values <0.05 were considered statistically significant. DM: diabetes; HTN: hypertension

Confounding Factors		Overall (n = 200)	Nosocomial infections		P-value
			Absent	Present	
Gender	Female	60 (30%)	50 (85%)	9 (15%)	0.13
	Male	140 (70%)	129 (92%)	11 (8%)	
Age	Adults (≥18 years)	138 (69%)	133 (96%)	5 (4%)	<0.003
	Older adults (≥65 years)	62 (31%)	47 (76%)	15 (24%)	
Subjective Global Assessment index	Well-nourished	101 (51%)	99 (98%)	2 (2%)	<0.001
	Undernourished	99 (49%)	81 (82%)	18 (18%)	
Serum albumin	Normal (≥3.5 g/dL)	133 (67%)	124 (93%)	9 (7%)	0.12
	Abnormal (<3.5 g/dL)	67 (34%)	56 (83%)	11 (16%)	
Comorbidities	None	116 (58%)	109 (94%)	7 (6%)	0.01
	DM	16 (8%)	16 (100%)	0	
	HTN	26 (13%)	23 (88%)	3 (12%)	
	Both DM and HTN	42 (21%)	32 (76%)	10 (24%)	

Distribution of nosocomial infections

**Figure 1:**
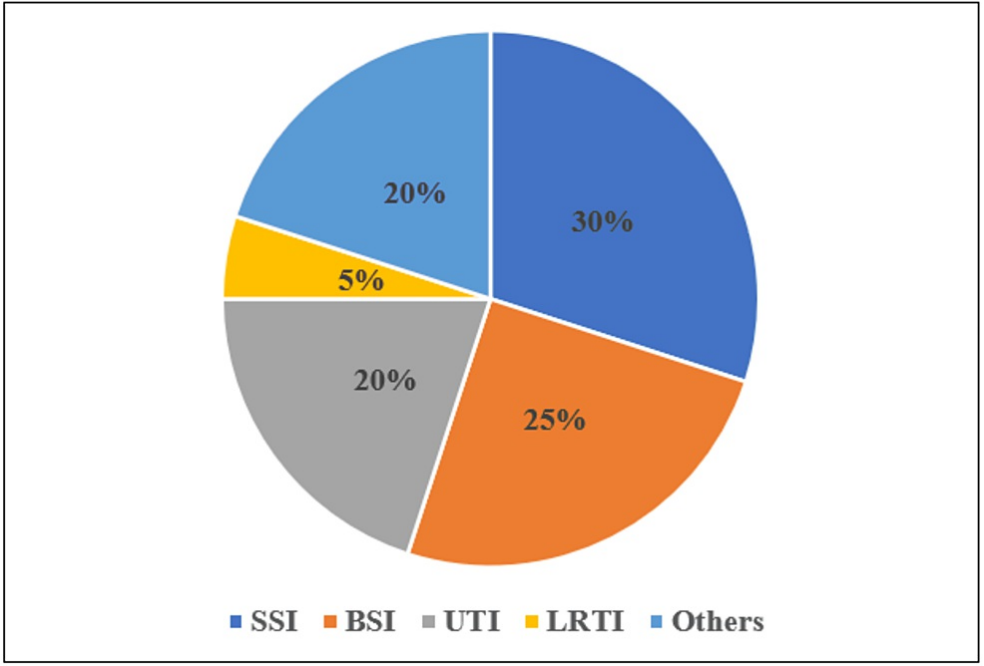
Distribution of types of nosocomial infections. BSI: bloodstream infection; SSI: surgical site infection; UTI: urinary tract infection; LRTI: lower respiratory tract infection; others: tracheal infection, biliary tract infection, bed sore, and respiratory infection

Among 200 patients 20 (10%) had NIs (p < 0.001). Surgical site infection (SSI) (6, 30%) and bloodstream infection (5, 25%) were the most common NIs seen in this study, followed by urinary tract infection (UTI) (4, 20%), lower respiratory tract infection (LRTI) (1, 5%), and others (tracheal infection, biliary tract infection, bed sore, and respiratory infection) (4, 20%) (Figure 1). It was found that of 20 NI cases, 15 (75%) were due to one or more than one invasive device. Among the pathogens isolated, *Klebsiella *was the predominant organism (Table 2).

**Table 2:** Distribution of NIs. Note: P-values <0.05 were considered statistically significant. NI: nosocomial infection

NI signs and symptoms	Overall (n = 200)	Incidence of NIs		P-value
		Absent	Present	
Absent	151 (76%)	149 (99%)	2 (1%)	<0.001
Present	49 (25%)	31 (63%)	18 (37%)	

Association between nutritional status and nosocomial infections

As shown in Table 3, the univariate relative risk (uRR) for the undernourished group was more clinically significant than the well-nourished group. The risk of getting NIs in the undernourished group was 6.10 times higher compared to the well-nourished group. Similarly, the result for uRR for DM and HTN together was clinically significant than having any one of the comorbidities. The risk of getting NIs in the group of both comorbidities, i.e., DM and HTN, together was 3.45 times higher compared to those who did not have any comorbidities.

**Table 3:** Effect of exposure variables on NI estimation of univariable relative risks. Note: n = 20; p-value <0.05 was considered statistically significant. The relative risk (RR) of greater than 2.0 was considered clinically significant. NI: nosocomial infection; SGA: subjective global index; DM: diabetes mellitus; HTN: hypertension; uRR: univariate relative risk; Ref: reference

Confounding factors	NIs, n (%)	Univariable analysis		
		uRR (95% CI)	P-value	
SGA index				
Well-nourished	2 (2%)	Ref.	-	
Undernourished	18 (18%)	6.10 (1.42–26.3)	0.02	
Comorbidities	7 (6%)	Ref.	-	
None				
DM	0	NA	-	
HTN	3 (12%)	1.80 (0.46–6.95)	0.40	
Both DM and HTN	10 (24%)	3.45 (1.31–9.07)	0.01	

As shown in Table 4, older adults (n = 5) were more likely to develop NIs than adults (n = 15) (24.2% to 3.6%). Participants with abnormal serum albumin levels (n = 11) were more prone to have NIs compared to participants falling in the normal range of serum albumin (n = 9) (16.4% to 6.8%).

**Table 4:** Percentage distribution of confounding variables for estimating the risk of getting NI. Note: n = 200. NI: nosocomial infection

Confounding variables			Nosocomial infections		Total
			Present	Absent	
Age	≥18 years (adults)	Count	5	133	138
		% within age	3.6%	96.4%	100.0%
	≥65 years (older adults)	Count	15	47	62
		% within age	24.2%	75.8%	100.0%
Total		Count	20	180	200
		% within age	10.0%	90.0%	100.0%
Serum albumin (g/dL)	Normal (≥3.5 g/dL)	Count	9	124	133
		% within serum albumin (g/dL)	6.8%	93.2%	100.0%
	Abnormal (<3.5g/dL)	Count	11	56	67
		% within serum albumin (g/dL)	16.4%	83.6%	100.0%
Total		Count	20	180	200
		% within serum albumin (g/dL)	10.0%	90.0%	100.0%

**Figure 2:**
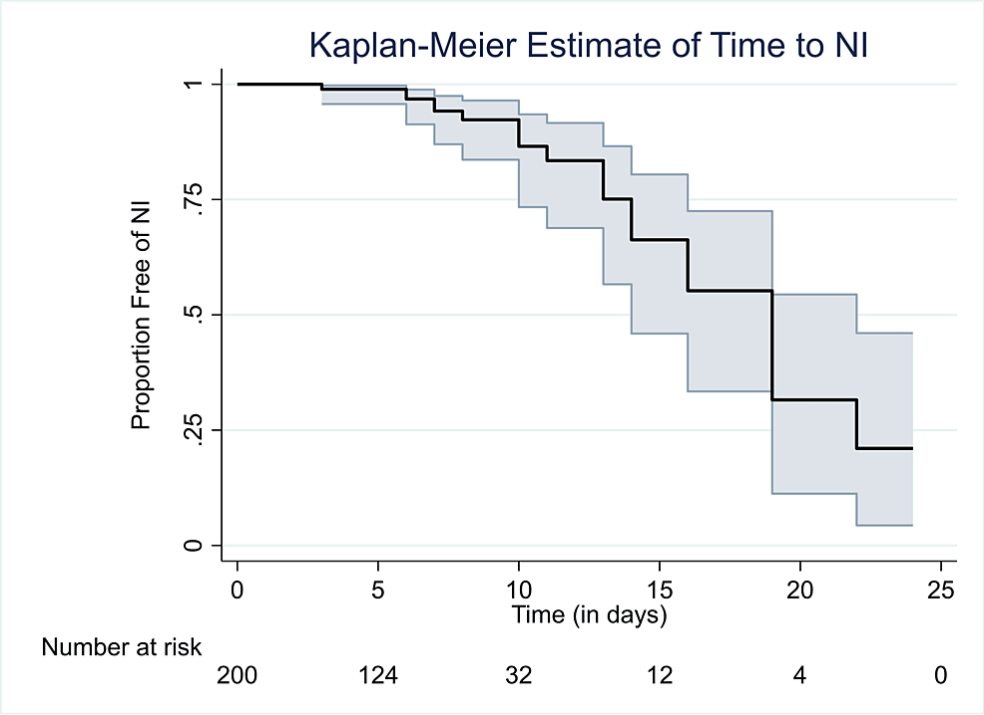
Time to event for rate of nosocomial infections. NI: nosocomial infection

**Figure 3:**
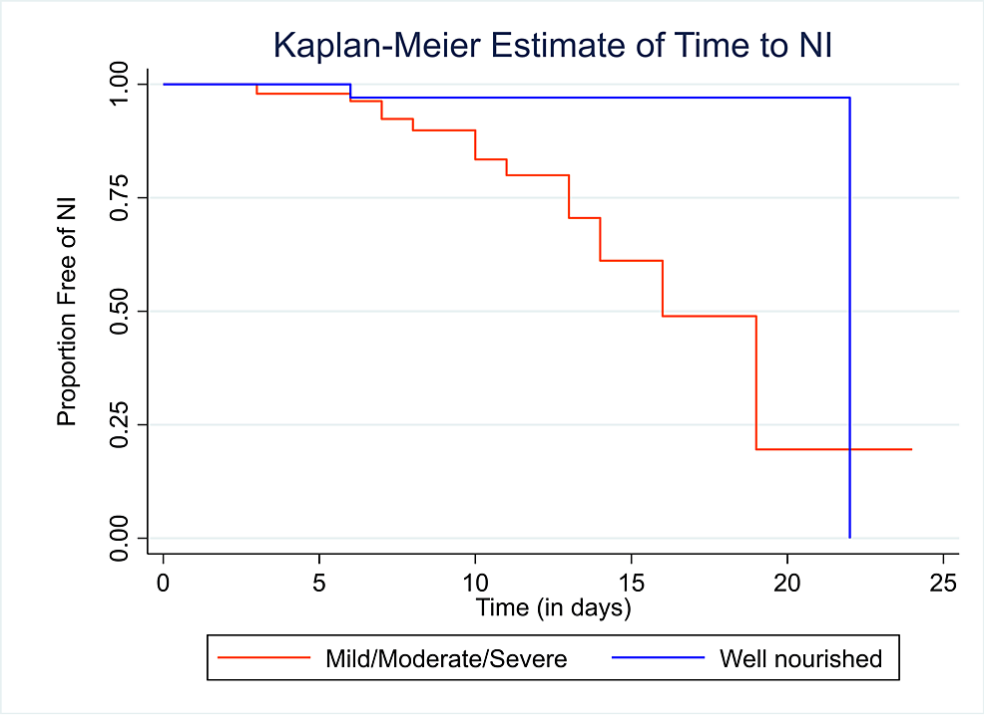
Time to event rate of nosocomial infections stratified by nutritional status. NI: nosocomial infection

According to the time to event for the rate of NIs, the overall survival time was 18 days, and with the passage of time, which was by the end of the follow-up of around 25 days, the chances of developing NIs had increased (Figure 2). In Figure 3, over time, the well-nourished group was less likely to develop NIs compared to the undernourished group.

## Discussion

This cohort study aimed to add to the existing data in the relevant field. This is an important topic of research as the risk of getting infections in the hospital setup leads to further direct and indirect exposure to the conditions of patients.

Distribution of nosocomial infections

In this study, most patients who developed NIs were on more than one invasive device or had undergone a medical procedure. Patients undergoing surgery were more prone to getting NIs which is similar to the findings of another study [7] which suggested that patients who underwent surgery were 2.35 times more likely to get NI. In addition to this, as per one case-control study [8] conducted on geriatric patients found that recent surgery was one of the risk factors for developing NIs. Therefore, in line with these findings, the predominate NI found in this study was SSI 6 (30%) which is correlated with previous study findings showing the incidence of SSI was 7.3% (range = 1.7-10.4%) with longer hospital stays than those who did not have SSI [9]. In the study by Scherbaum et al. (2014), among other NIs, SSI was the most common (20, 44%) [10]. Another prospective study involving 1,637 patients showed the surgical site to be the second most common site for NI [2].

The most common pathogen isolated in this study was *Klebsiella*. According to one systemic review conducted by Chisti et al. (2009), among the other commonly isolated bacterial pathogens, *Klebsiella pneumoniae* was the most common cause of pneumonia in severely malnourished children [11]. Though *Klebsiella *species shows no geographical variation in frequency, it accounts for approximately 8% of all hospital-acquired infections [12]. Hence, *Klebsiella *remains a burden on the economy and the life expectancy of patients. Many preventive measures have been taken to control these infections but with time new approaches need to be included with a scientific backdrop against *Klebsiella *infections.

Association between nutritional status and nosocomial infections

In this study, the poor nutritional status of the patients has been taken as a risk factor for developing NIs. According to our study, undernourished patients had more chances of acquiring NI (uRR = 6.10, 95% CI) than well-nourished patients. This is similar to the findings of Aiken et al. (2011) who conducted a prospective cohort study to investigate the prevalence and cause of hospital-acquired infections in children in the Kilifi District of Africa. They found that nosocomial bacteremia was significantly associated with severe malnutrition (hazard ratio = 2.52, 95% CI = 1.79-3.57) [13]. The study conducted in an intensive care unit by Lee et al. (2003) reported that severely malnourished patients were more likely to develop NI (2.1 times for total infection) than well-nourished patients [14]. They also combined serum albumin as one of the nutritional markers with another subjective method of nutritional assessment as there is no one standard method of determining nutritional status. Albumin is considered a good variable for patients’ underlying disease conditions because it is a combination of a biological marker related to body composition and acute-phase response. Therefore, it is generally not used to assess nutritional status [15] as it may vary in different conditions such as hepatic and renal failure and systematic inflammatory response (inflammation, injury, infection) [16-18].

Therefore, to draw more comprehensive outcomes different objective and subjective methods should be used together for nutritional assessment. This might increase the specificity and sensitivity of diagnosing malnourishment, as reported by Schneider et al. (2004) [2]. In their study, they combined nutritional risk index (NRI) and serum albumin to evaluate nutritional status and found that the prevalence of NI was 4.4% in non-malnourished patients, 7.6% in moderately malnourished patients, and 14.6% in severely malnourished patients (chi-square test, p = 0.009). In univariate analysis, the odds ratios for NI were 1.46 (95% CI = 1.2, 2.1) in moderately malnourished patients and 4.98 (95% CI = 4.6, 6.4) in severely malnourished patients. They also found that in univariate analysis serum albumin levels (r^2^ = 0.04, p = 0.04) were associated with NI. Hence, like our study, they considered malnourishment as one of the risk factors in developing NIs.

Additional risk factors for the development of nosocomial infections

Age

According to the WHO, older age comprises those ≥65 years of age. It is estimated that by the year 2025, the world’s elderly population will exceed 800 million, with females accounting for most of them, and about 20% of the world population will be ≥65 years old by 2050 [19,20].

Advanced age leads to many health problems. In the elderly, aging leads to immune system change, tissue organ change, chronic diseases, malnutrition, and functional deficiencies (immobilization, incontinence, dysphagia) [21].

Several studies have shown aging to be an associated risk factor for the development of NI. In this study, there was a significant relationship between age and NI (p = 0.003). In the study conducted by Leone et al. (2003), advanced age is closely related to NI [22]. Similarly, Richards et al. (1999) found that aging was a risk factor for NI and mortality [23].

Several precautions such as the minimum use of invasive procedures and reducing unnecessary medical intervention may protect these age groups from the risk of NI as they are already immunologically and physiologically compromised.

Comorbidities

DM and HTN are a gateway to many diseases. Diabetic patients will increase to 366 million by 2030 and adults with HTN are predicted to increase by 60% to a total of 1.56 billion by 2025. These two metabolic conditions are usually intertwined together which alone gives the path to many complications [24,25].

Our study showed participants with both comorbidities together have higher chances of getting NI (uRR = 3.45, 95% CI). According to the study by Levin et al. (2011), HTN and DM were the individual risk factors for SSI [26]. The study by Durgad et al. (2017) revealed that DM was a risk factor for UTI [27]. Similarly, Dubory et al. (2015) found that the presence of diabetes is associated with SSI [28].

Limitations of the study

This study has developed a comprehensive method of assessing nutritional status and evaluating NIs in hospitalized adults. However, the study also has some limitations. First, this study was conducted during the COVID-19 pandemic due to which it was only possible to include a limited number of participants. Second, the study used a one-day nutritional assessment and did not assess prospective nutritional status for the entire hospital stay. This would have helped in seeing changes in nutritional status (improvement vs. worsening) and its impact on clinical outcomes.

## Conclusions

Though NI is seen globally, in developing countries, studies related to NIs are scarce. This cohort study revealed a high incidence of NI likely because it included almost all wards of the hospital and varied age distribution (≥18 years). The findings of this study reveal a greater incidence of infection in the malnourished group; they were 6.1 times more prone to getting NI than the well-nourished group. This finding suggests that poor nutritional status during hospitalization can be a risk factor for NI. Future studies can opt for early nutritional assessment during the time of admission to reduce the incidence of NI. As tools used for assessing nutritional status are subjective, this should be combined with any of the serum markers used in nutritional screening to justify the outcome in a more comprehensive way.
